# Diagnostic value of cardiac magnetic resonance imaginng in patients with suspected acute myocardial infarction without significant coronary stenosis

**DOI:** 10.1186/1532-429X-17-S1-P153

**Published:** 2015-02-03

**Authors:** Annachiara Aldrovandi, Filippo Pigazzani, Diego Ardissino

**Affiliations:** Division of Cardiology, Azienda Ospedaliero-Universitaria di Parma. Italy, Parma, Italy

## Background

The aim of the study is to evaluate the incremental diagnostic value of cardiac magnetic resonance (CMR) in patients (pts) with suspected acute myocardial infarction (AMI) without significant coronary stenosis at coronary angiography.

## Methods

One hundred forty six consecutive pts (age 61 ± 14, 83 [57%]) females) with suspected AMI and normal coronary arteries or nonobstructive coronary artery disease at coronary angiography were enrolled. Suspected AMI was defined as typical chest pain lasting >20', electrocardiographic (EKG) changes and increased cardiac enzymes (troponin I>99th percentile and creatine kinase MB levels more than twice the upper normal limit). All pts underwent CMR 6.2 ± 5.7 days after admission using a 1.5T MRI system with cine imaging with breath-hold steady-state free-precession sequences, T2 imaging and late enhancement imaging after gadolinium injection (LGE). The image analysis included: presence of myocardial edema on T2 images; presence, localization and pattern of distribution of LGE.

## Results

The admission EKG showed ST elevation in 34 pts (23%). Median peak troponin I and peak CK MB values were 6.5 ± 13.8 ng/ml and 32.2 ± 49.2 ng/ml respectively. Mean ejection fraction was 53% ± 10%. LGE was present in 64 (45%) pts and edema in 41 (28%) pts. LGE distribution was anterior in 27 pts, lateral in 28 pts and inferior in 30 pts. Peak Troponin I value was significantly higher in pts with LGE on CMR imaging compared to those without LGE (9.9 ± 16.1 ng/ml vs 3.8 ± 11.4 ng/ml) After analyis of CMR images, an acute myocardial infarction was confirmed in 42 pts, while CMR findings suggested myocarditis in 20 pts, apical ballooning in 20 pts, hypertrophic cardiomyopathy in 3 pts.

## Conclusions

CMR provides incremental diagnostic value in a population of pts with suspected acute coronary syndrome without obstructive coronary stenosis at coronary angiography, either by confirming the initial diagnosis or suggesting a different diagnosis. However a portion of patients do not show any LGE at CMR, probably due to the low level of released Troponin I.

## Funding

No funding.

